# Super-refractory Status Epilepticus in Febrile Infection-Related Epilepsy Syndrome Triggered by Influenza A: A Pediatric Case Report

**DOI:** 10.7759/cureus.112092

**Published:** 2026-07-05

**Authors:** Malik Ocakdan

**Affiliations:** 1 Intensive Care Unit, Cliniques Universitaires Saint-Luc, Brussel, BEL

**Keywords:** claustrum sign, cytokines, febrile infection-related epilepsy syndrome, immunotherapy, influenza a, pediatric critical care, super-refractory status epilepticus

## Abstract

Febrile infection-related epilepsy syndrome (FIRES) is a rare cause of new-onset refractory status epilepticus following a febrile illness. We report the case of a previously healthy three-year-old boy who developed focal seizures in the context of a confirmed Influenza A infection, rapidly progressing to super-refractory status epilepticus.

Initial investigations, including cerebrospinal fluid analysis and neuroimaging, were unremarkable. Despite early multimodal therapy with multiple antiseizure medications, continuous anesthetic infusions, immunomodulatory treatments, and a ketogenic diet, seizure control remained transient. Continuous EEG confirmed persistent super-refractory status epilepticus, while cytokine analysis demonstrated elevated interleukin-6 and interleukin-8 levels. Follow-up MRI showed bilateral claustrum and hippocampal involvement, later progressing to diffuse cerebral atrophy.

Despite escalation of therapy, including intrathecal corticosteroids and vagal nerve stimulation, the patient showed no sustained improvement. Care was ultimately redirected toward palliation, and the patient died after 50 days of intensive care.

This case highlights the fulminant and refractory nature of FIRES, supports a potential role of cytokine-driven neuroinflammation, and suggests a possible association with a preceding viral infection.

## Introduction

Febrile infection-related epilepsy syndrome (FIRES), a subtype of new-onset refractory status epilepticus (NORSE), is a rare and life-threatening neurological disorder affecting both children and adults [1]. Although uncommon, FIRES is associated with mortality rates approaching 30% and severe long-term neurological sequelae, including cognitive impairment in the majority of survivors, making it one of the most devastating forms of status epilepticus [2].

The pathophysiology of FIRES remains incompletely understood. Current evidence supports a neuroinflammatory mechanism, potentially triggered by a preceding infectious event, leading to a marked reduction in seizure threshold and the development of refractory status epilepticus [2-4]. This hypothesis is supported by the characteristic biphasic clinical presentation, in which a nonspecific febrile illness precedes the onset of status epilepticus by 24 hours to two weeks, as well as by studies demonstrating elevated pro-inflammatory cytokines and activation of both innate and adaptive immune pathways in the cerebrospinal fluid and serum of affected patients [2-4]. Despite these advances, no specific biomarker currently exists to allow early diagnosis, which remains primarily clinical.

Management of FIRES is particularly challenging because of its frequently super-refractory course. Current international recommendations advocate a multimodal therapeutic approach combining antiseizure medications, anesthetic agents, immunomodulatory therapies such as tocilizumab and anakinra, and a ketogenic diet [1]. However, response to treatment is often limited, resulting in prolonged intensive care unit stays requiring mechanical ventilation and exposing patients to numerous treatment-related and ICU-associated complications.

We report the case of a previously healthy three-year-old boy who developed febrile seizures in the context of a confirmed Influenza A infection before rapidly progressing to super-refractory status epilepticus. This case highlights the diagnostic and therapeutic challenges associated with FIRES, illustrates the limitations of currently available treatments, and further supports the hypothesis of a post-infectious neuroinflammatory mechanism.

## Case presentation

A previously healthy three-year-old boy was brought to the emergency department by the prehospital team after developing seizures at home following a four-day history of intermittent fever. Four days before admission, he developed a fever of up to 38.8 °C without associated symptoms. The fever persisted for 48 hours, followed by a 24-hour afebrile period, before recurring on the day of admission, accompanied by anorexia and fatigue. Around noon on the day of admission, his mother observed an episode of altered consciousness accompanied by hypersalivation and hypotonia, prompting activation of emergency medical services.

Upon arrival of the prehospital medical team, the patient exhibited clonic movements of the right upper limb associated with rightward gaze deviation. Oxygen saturation was 94%, and capillary blood glucose was within normal limits. Intravenous midazolam (1.5 mg followed by an additional 0.5 mg) resulted in partial but incomplete resolution of seizure activity. Upon arrival at the emergency department, the patient was no longer seizing but remained in a post-ictal state. Vital signs showed a temperature of 37.5 °C, heart rate of 124 beats/minute, respiratory rate of 25 breaths/minute, blood pressure of 69/42 mmHg, and oxygen saturation of 99% on a non-rebreather mask at 15 L/minute. Neurological examination revealed an altered level of consciousness with a Glasgow Coma Scale score of 7/15 (E1V2M4). Pupils were equal, round, and reactive to light. Transient divergent strabismus was noted, although subsequent examinations revealed no cranial nerve abnormalities. There was no nuchal rigidity or nystagmus, and no focal motor deficits were identified. Cardiopulmonary examination was unremarkable, with normal heart sounds and symmetrical air entry without respiratory distress. Peripheral perfusion was preserved, with a capillary refill time of 2 seconds and no mottling. The abdomen was soft and non-tender without organomegaly. Otolaryngological examination revealed an erythematous and congested left tympanic membrane, together with pharyngeal erythema. No petechiae were observed.

Initial laboratory investigations are summarized in Table 1. A nasopharyngeal swab was positive for Influenza A.

**Table 1:** Initial lab results in the ED. ED, emergency department; PCR, polymerase chain reaction; TMA, thrombotic microangiopathy; eGFR, estimated glomerular filtration rate; MCV, mean corpuscular volume; IDMS, isotope dilution mass spectrometry

Serum markers	References	Results
Hemoglobin (g/dL)	10.2-12.7	10.9
Hematocrit (%)	38%-48%	35
RBC count	3.89-4.97 × 10e6/µL	4.22
MCV (fL)	71.3-84.0	83.2
WBC count	4.90-12.90 × 10³/µL	9.27
Neutrophils	1.50-6.90 × 10³/µL	7.05
Lymphocytes	1.70-5.50 × 10³/µL	1.47
Monocytes	0.15-1.28 × 10³/µL	0.42
Eosinophils	0.04-1.19 × 10³/µL	0.00
Basophils	0.02-0.12 × 10³/µL	0.00
Ratio neutron/lympho	0.78-3.53	4.80
Neutrophils (%)	40.0-70.0	76.1
Lymphocytes (%)	20.0-50.0	15.9
Monocytes (%)	5.0-10.0	4.5
Eosinophils (%)	0.5-6.0	0.0
Basophils (%)	0.1-2.0	0.0
Myelemia (%)	<=2.00	0
Reactional lymphocytes	0.01-0.01 × 10³/µL	0.32
Reactional lymphocytes (%)	0.1-0.1	3.5
Platelets	189-403 × 10³/µL	332
CRP (mg/L)	<=5.0	11.3
Creatinine (IDMS) (mg/dL)	0.60-1.30	0.47
eGFR	>=75 mL/minute/1.73m²	73
Sodium (mmol/L)	135-145	137
Potassium (mmol/L)	3.50-5.00	3.72
Chloride (mmol/L)	97-107	98
Bicarbonate (mmol/L)	22.0-29.0	23.7
Calcium total (mmol/L)	2.20-2.70	2.24
Total plasma protein (g/L)	60-80	68
COVID-19 (PCR/TMA)	-	Not detected
PCR Influenza A	-	Positive
PCR Influenza B	-	Not detected
PCR Respiratory syncytial virus	-	Not detected

During his stay in the emergency department, the patient developed recurrent focal seizures characterized by clonic movements of the right upper limb. He was treated with intravenous midazolam (0.1 mg/kg), followed by a levetiracetam bolus (40 mg/kg). In the context of febrile seizures and concern for central nervous system infection, empirical treatment with ceftriaxone (80 mg/kg) and acyclovir (15 mg/kg) was initiated. A 10-day course of oseltamivir was also started.

A brain CT scan was performed due to the prolonged post-ictal state and concern for intracranial pathology, and showed no evidence of space-occupying lesions. A lumbar puncture revealed clear cerebrospinal fluid (CSF) with normal opening pressure. CSF results are summarized in Table 2.

**Table 2:** Cerebrospinal fluid (CSF) analysis in the ED. NMDA, N-methyl-D-aspartate; MOG, myelin oligodendrocyte glycoprotein; ED, emergency department

CSF markers	References	Results
Total nucleated cells (/µL)	<=20/µL	2
WBC (/µL)		2
Polymorphonuclear (/µL)		0
Mononuclear cells (/µL)		2
RBC	10³/µL	1
Neutrophils		12
Lymphocytes		52
Monocytes/macrophages		36
Glucose (mg/dL)	40-80	98
Lactate (mmol/L)	1.1-2.4	1.1
Proteins (mg/dL)	15-45	55
Anti-NMDA antibodies		Negative
Anti-MOG antibodies		Negative
CSF culture		Culture negative

The patient was subsequently admitted to the pediatric intensive care unit for further management.

Upon admission to the pediatric intensive care unit, the patient required intubation and mechanical ventilation due to persistent seizures and the need for continuous sedation with midazolam and propofol. Continuous EEG monitoring demonstrated ongoing epileptic activity, initially predominantly involving the left temporal lobe, later evolving into bilateral generalized seizure activity (Figure 1).

**Figure 1:**
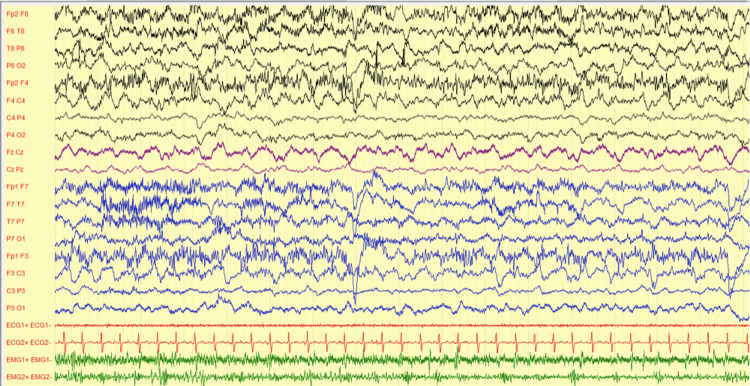
Continuous EEG monitoring on Day 1 of ICU. Continuous EEG demonstrated rhythmic, evolving epileptiform activity arising predominantly from the left temporal region with bilateral propagation, consistent with focal status epilepticus (F7-T7 and T7-P7 channels).

Despite treatment with multiple antiseizure medications, including levetiracetam, phenytoin, valproic acid, lacosamide, topiramate, and phenobarbital, seizure control was not achieved. Continuous infusions of midazolam and ketamine were initiated, followed by propofol, which was subsequently discontinued because of a lack of efficacy. Early immunomodulatory therapy consisted of high-dose methylprednisolone (30 mg/kg/day for five days), intravenous immunoglobulins, and tocilizumab administered on Days 5 and 6 of admission. Because C-reactive protein levels cannot be reliably interpreted following anti-IL-6 therapy, daily blood cultures were performed for infectious surveillance. Active temperature management using Criticool was initiated to maintain a core temperature of 35-36 °C, while a ketogenic diet was introduced and progressively optimized. Diagnostic investigations were initially unrevealing. Brain MRI performed on Day 2 was normal, whereas repeat imaging on Day 9 demonstrated symmetrical claustral hyperintensities and bilateral hippocampal involvement, findings consistent with FIRES. CSF analysis showed no evidence of infection, including negative viral studies for herpes simplex virus type 1 (HSV-1) and herpes simplex virus type 2 (HSV-2), and autoimmune encephalitis antibodies were absent (Table 2). Extensive metabolic and genetic investigations, including next-generation sequencing of an encephalopathy panel, were inconclusive. However, CSF neurofilament levels were markedly elevated (12,714 pg/mL; normal <1,000 pg/mL), suggesting significant axonal injury and neuronal damage.

Given the persistence of super-refractory status epilepticus, continuous thiopental infusion was initiated, resulting in EEG burst suppression and temporary seizure control between Days 6 and 8. Thiopental-induced vasoplegia required continuous low-dose norepinephrine support (maximum 0.06 mcg/kg/minute). Because of the ongoing refractory course, anakinra was introduced on Day 10. Empirical antimicrobial therapy with ceftriaxone and acyclovir was discontinued after a negative infectious workup, while prophylactic cotrimoxazole was started in the context of escalating immunosuppressive therapy. A single blood culture grew Staphylococcus epidermidis, prompting brief treatment with intravenous vancomycin; however, repeat cultures remained negative, and the isolate was considered a contaminant. Subsequent microbiological analyses of endotracheal aspirates and urine samples identified multidrug-resistant organisms, including Pseudomonas aeruginosa, Acinetobacter baumannii, and carbapenem-resistant Enterobacter cloacae. Although transient antimicrobial therapy was initiated, the absence of clinical or biological evidence of infection, together with negative repeat cultures, ultimately supported colonization rather than active infection, highlighting the risk of multidrug-resistant organism colonization during prolonged intensive care stays.

Despite this aggressive multimodal approach, the patient remained in super-refractory status epilepticus. Although transient seizure control was achieved under thiopental-induced burst suppression, repeated attempts to wean anesthetic therapy were followed by recurrent epileptic activity and multiple relapses (Figure 2).

**Figure 2:**
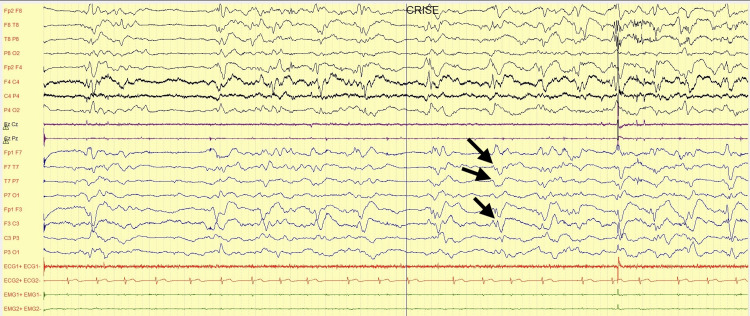
Representative EEG recording obtained following thiopental weaning. Representative EEG recording obtained after thiopental weaning. The vertical marker indicates seizure activity. Bilateral spike-wave discharges are visible, with a clear predominance over the left temporocentral region (arrows), consistent with recurrent epileptic activity following the loss of burst suppression.

Anti-seizure treatment was further escalated with the introduction of perampanel, later complemented by pregabalin, vigabatrin, and brivaracetam. Several agents were subsequently discontinued because of lack of efficacy or adverse effects, including felbamate, which was stopped because of hepatotoxicity. Recurrent seizure activity necessitated repeated thiopental boluses and intermittent reintroduction of continuous anesthetic infusions, including midazolam and ketamine. In parallel, immunomodulatory therapy was intensified. A second course of tocilizumab was administered, and anakinra was continued for several weeks. Intrathecal methylprednisolone was given in six doses, while intravenous immunoglobulins were reintroduced as a bridging strategy before planned cyclophosphamide therapy. Cyclophosphamide was ultimately not initiated following a multidisciplinary decision to de-escalate treatment during the final days of hospitalization. Rituximab was also considered as a second-line immunotherapy; however, the absence of autoantibodies in the CSF argued against its use.

Despite these interventions, neuroimaging demonstrated progressive disease evolution. While the initial MRI was unremarkable, follow-up examinations revealed diffuse cortical thickening predominantly involving the insular and temporal regions, together with bilateral claustral and hippocampal abnormalities characteristic of FIRES. Subsequent imaging demonstrated diffuse cerebral atrophy associated with global hypoperfusion, findings consistent with severe ongoing neurological injury (Figures 3-4).

**Figure 3:**
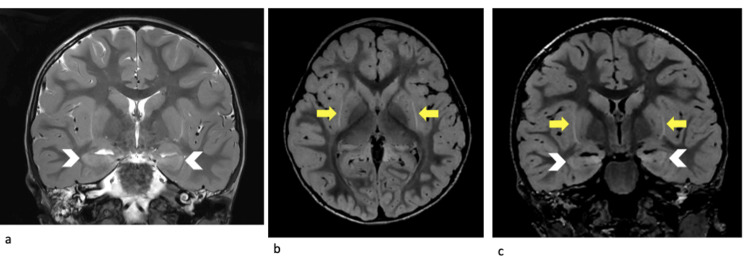
Brain MRI on Day 9. Brain MRI obtained on day 9. Coronal T2-weighted (a), axial FLAIR (b), and coronal FLAIR (c) images demonstrate diffuse cortical thickening, predominantly involving the insula and temporal lobes. Bilateral T2 and FLAIR signal hyperintensity in the hippocampal cortex (arrowheads in a and c); bilateral hyperintensity of the claustrum ("claustrum sign") (yellow arrows in b and c). FLAIR, fluid-attenuated inversion recovery

**Figure 4:**
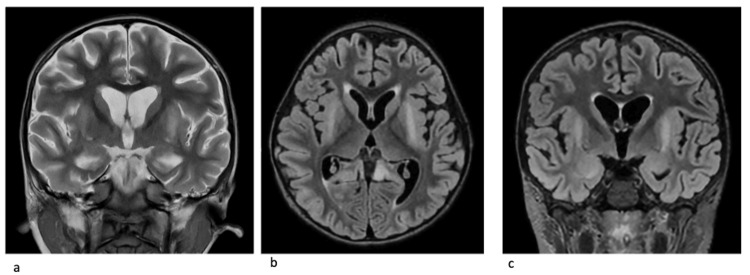
Brain MRI on Day 30. A brain MRI was performed three weeks later. Coronal T2-weighted (a), axial FLAIR (b), and coronal FLAIR (c) images demonstrate significant diffuse cortical and subcortical volume loss. Persistent bilateral claustral hyperintensity is also evident. FLAIR, fluid-attenuated inversion recovery

CSF cytokine analysis was sent to Paris and showed high levels of IL-6 and IL-8, as well as evidence of Th1/Th17 activation in serum and CSF (Table 3).

**Table 3:** Serum and cerebrospinal fluid (CSF) cytokine analysis. G-CSF, granulocyte colony-stimulating factor; IL-1β, interleukin-1β; IL-4, interleukin-4; IL-6, interleukin-6; IL-8, interleukin-8; IL-10, interleukin-10; IL-17A, interleukin-17A; MIP-1α, macrophage inflammatory protein-1α; TNF-α, tumor necrosis factor-α; IL-12p70, interleukin-12p70; IL-2, interleukin-2

Cytokine	Serum (pg/mL)	Mean normal(pg/mL)	Upper limit normal (pg/mL)	CSF (pg/mL)	Mean normal (pg/mL)	Upper limit normal (pg/mL)
GCSF	0.49	0.15	0.44	2.57	0.052	0.24
IL1b	0.08	Not detectable	Not detectable	0.02	Not detectable	Not detectable
IL4	2.31	0.15	1.01	Not detectable	Not detectable	Not detectable
IL6	26.88	4.05	9.92	174.09	8.60	37.71
IL8	25.65	19.54	65.43	469.53	6.12	16.46
IL10	9.90	0.44	2.33	7.18	0.072	0.28
IL17A	6.54	0.090	0.70	0.70	0.027	0.17
MIP-1α	2.81	4.59	23.89	26.40	0.092	0.41
TNFa	6.49	0.061	0.38	4.89	Not detectable	Not detectable
IL12p70	18.17	0.18	0.83	3.13	Not detectable	Not detectable
IL2	3.15	1.51	6.23	1.09	0.030	0.20

Serial CSF analyses demonstrated persistent elevation of interleukin-6 (IL-6), while other inflammatory markers remained largely unremarkable. Additional adjunctive therapies included implantation of a vagal nerve stimulator and magnesium sulfate infusion targeting therapeutic serum concentrations of 2-4 mmol/L. Despite these interventions, seizure control remained incomplete. Given the persistent super-refractory status epilepticus, a pragmatic approach was adopted whereby a predefined threshold of electrographic seizure burden was accepted to limit the adverse effects associated with prolonged anesthetic coma.

The clinical course was further complicated by treatment-related toxicity. Significant hepatotoxicity, attributed to anakinra and felbamate, was observed, with peak liver function test values of gamma-glutamyl transferase (GGT) 3,700 U/L, alanine aminotransferase (ALT) 395 U/L, aspartate aminotransferase (AST) 434 U/L, and total bilirubin 1.4 mg/dL. The patient also developed ultrasound-confirmed caudocorporeal pancreatitis (peak lipase 933 U/L), probably secondary to severe hypertriglyceridemia (peak 4,782 mg/dL) in the context of ketogenic diet therapy, as well as felbamate-associated metabolic acidosis, prompting discontinuation of the implicated treatments. While a ketogenic diet is a recognized cause of hypertriglyceridemia and pancreatitis, the contribution of concomitant therapies cannot be determined in this case. The need to balance seizure control against treatment-related toxicity, prolonged deep sedation, and hemodynamic instability added considerable complexity to management. Following multidisciplinary discussions involving pediatric intensive care and neurology teams, the prognosis was considered extremely poor because of the prolonged disease course, resistance to maximal therapy, and progressive cerebral atrophy on neuroimaging. In agreement with the patient's parents, a multidisciplinary decision was made to limit further escalation of care and prioritize comfort measures given the extremely poor neurological prognosis. As part of this approach, the patient was extubated to assess his ability to maintain spontaneous ventilation and protect his airway. Immediately after extubation, he was unable to adequately clear his oral secretions, resulting in aspiration, rapid oxygen desaturation, and severe hypoxemic respiratory failure. Given the previously established goals of care, re-intubation was not pursued. Palliative sedation was initiated to ensure comfort, and the patient died peacefully later that day in the presence of his parents.

A schematic overview of the patient's clinical course, treatments, EEG evolution, and neuroimaging findings is provided in Figure 5.

**Figure 5:**
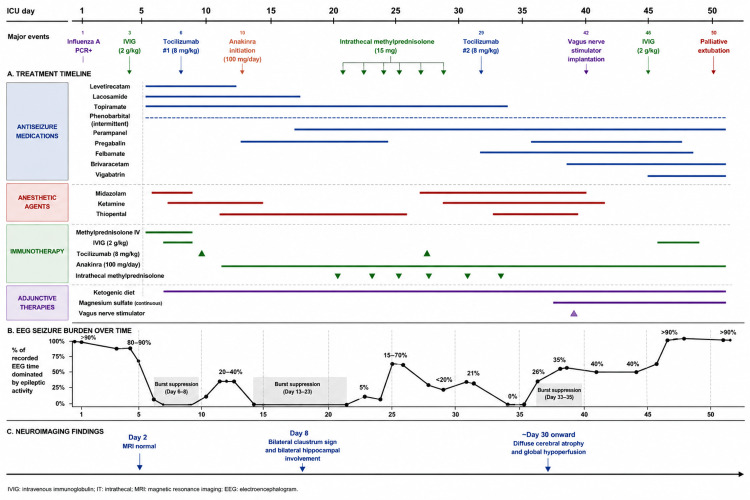
Clinical timeline infographic. Temporal evolution of treatment, EEG seizure burden, and neuroimaging findings during the 50-day PICU stay. The upper panel shows major therapeutic interventions, including antiseizure medications, anesthetic agents, immunotherapies, and adjunctive treatments. The middle panel illustrates the percentage of recorded EEG time occupied by epileptic activity, highlighting periods of burst suppression and subsequent relapses. The lower panel summarizes MRI findings, demonstrating progression from a normal initial MRI to bilateral claustral and hippocampal involvement, followed by diffuse cerebral atrophy and global hypoperfusion. Despite multimodal therapy, seizure control remained transient, with progression to super-refractory status epilepticus. EEG, electroencephalography; MRI, magnetic resonance imaging; PICU, pediatric intensive care unit

## Discussion

FIRES, a subgroup of NORSE, is a rare, complex, and devastating condition affecting both children and adults. It is defined as refractory status epilepticus without a clear acute structural, toxic, or metabolic cause, occurring between 24 hours and two weeks after a febrile illness [1].

The pathophysiology of FIRES remains incompletely understood. A post-infectious, immune-mediated mechanism with prominent neuroinflammation is currently the most widely accepted hypothesis. This is supported by elevated levels of pro-inflammatory cytokines in CSF and serum, particularly interleukin-1 (IL-1) and IL-6, compared to afebrile status epilepticus [2-4]. Furthermore, the reported efficacy of targeted immunotherapies, including anti-IL-1 and anti-IL-6 agents, in selected cases supports the role of cytokine-driven inflammation in disease pathogenesis [5-6]. FIRES carries a high mortality rate of up to 30% and is associated with significant long-term neurological sequelae in 66%-100% of survivors [2,7].

Our case, while typical in its clinical course, is notable for the identification of a preceding influenza A infection confirmed by polymerase chain reaction (PCR). The initial diagnostic workup was unrevealing, with normal CSF analysis, brain CT, and early MRI findings, consistent with previously reported cases [8]. Given the patient’s age, an extensive metabolic and genetic evaluation was performed, including analysis of genes associated with epileptic encephalopathies and mitochondrial disorders (DNM1, POLG, CACNA1A, KCNT1, SCN1A, SCN2A), all of which were negative in accordance with current recommendations [1]. Elevated neurofilament levels early in the course suggested significant axonal injury.

Continuous EEG monitoring allowed early confirmation of ongoing seizure activity, and the diagnosis of FIRES was considered on day one of admission. This prompted rapid escalation to a multimodal therapeutic approach including multiple antiseizure medications, continuous anesthetic infusions (midazolam, thiopental), immunomodulatory therapies (tocilizumab, anakinra), and initiation of a ketogenic diet. Cytokine profiling demonstrated increased pro-inflammatory activity with elevated IL-6 and IL-8 levels in both serum and CSF, while IL-1β elevation was modest. This cytokine pattern may partially explain the limited response to anakinra in this patient. We also observe an elevation in IL-10 levels, an anti-inflammatory cytokine, consistent with findings from other studies [1], and may reflect inefficient attempts to downregulate the inflammatory process.

Despite aggressive therapy, seizure control remained transient, with only brief periods of suppression before recurrence of status epilepticus. Follow-up MRI demonstrated characteristic bilateral claustrum and hippocampal involvement, followed by progressive diffuse cerebral atrophy, a finding associated with poor neurological outcomes in FIRES. Although the so-called claustrum sign is neither pathognomonic nor diagnostic of FIRES, it has also been described in other cytokine-mediated neuroinflammatory disorders, including COVID-19 encephalopathy and acute necrotizing encephalopathy. It has therefore been proposed to reflect widespread dysfunction of the cytokine-mediated neuroinflammatory network rather than to serve as a disease-specific imaging marker [9].

This case also illustrates the significant burden of prolonged intensive care management. Complications included hemodynamic instability related to deep anesthetic coma, requiring vasopressor support, as well as treatment-related adverse effects such as hypertriglyceridemia-induced pancreatitis associated with the ketogenic diet and hepatotoxicity related to antiseizure medications. These complications highlight the delicate balance between aggressive seizure control and treatment-related morbidity.

All therapeutic interventions were consistent with current international recommendations. Among all therapeutic interventions, anesthetic coma with thiopental was associated with the most consistent and reproducible electrographic seizure suppression, achieving burst suppression and transient seizure cessation on multiple occasions. However, seizure activity invariably recurred upon dose reduction or discontinuation. Other treatments, including multiple antiseizure medications, immunomodulatory therapies, ketogenic diet, and vagal nerve stimulation, did not result in sustained seizure control. Ketamine and Midazolam infusion were associated with transient improvement in a limited instance but lacked reproducibility. Notably, the limited efficacy of anakinra contrasted with elevated IL-6 levels but only mildly elevated IL-1 levels, suggesting a potential mismatch between cytokine profile and targeted immunotherapy. Rituximab was considered but ultimately not administered. Although seronegative NORSE/FIRES has been described, the absence of detectable neuronal autoantibodies made an antibody-mediated autoimmune encephalitis less likely, and the multidisciplinary team considered rituximab unlikely to provide additional benefit. Cannabidiol, though described as effective in a case series [10], was not used, as it was unavailable at the time of treatment. Surgical options were not considered due to the multifocal nature of seizure onset.

Although prolonged ICU courses with favorable outcomes have been reported, the absence of sustained seizure control, progressive cerebral atrophy as a major factor of poor prognosis [11], and accumulating treatment-related complications led to a multidisciplinary decision to limit further escalation of care. In agreement with the patient’s family, priority was given to quality of life. Following extubation, the patient developed rapid respiratory failure, and palliative sedation was initiated. He died shortly thereafter in the presence of his parents.

## Conclusions

FIRES is a rare but devastating neuroinflammatory disorder characterized by super-refractory status epilepticus and substantial mortality and neurological morbidity. This case highlights the remarkable therapeutic resistance of the disease, with persistent seizure activity despite extensive use of antiseizure medications, anesthetic agents, immunotherapies, ketogenic diet, and vagal nerve stimulation in accordance with current international recommendations.

The coexistence of a documented Influenza A infection and elevated inflammatory cytokines is consistent with a post-infectious neuroinflammatory process. However, this single case does not establish a causal relationship, and alternative triggers cannot be excluded. Further research is needed to better define the underlying mechanisms of FIRES, identify prognostic biomarkers, and develop more effective targeted therapeutic strategies.
